# Influence of statins in metastatic castration-resistant prostate cancer patients treated with new antiandrogen therapies: a systematic review and meta-analysis

**DOI:** 10.31744/einstein_journal/2022RW6339

**Published:** 2022-03-22

**Authors:** Renato Mariano, Kevin Lima Tavares, Renato Panhoca, Marcus Sadi

**Affiliations:** 1 Universidade de São Paulo São Paulo SP Brazil Universidade de São Paulo, São Paulo, SP, Brazil.; 2 Hospital do Servidor Púbico Estadual “Francisco Morato de Oliveira” São Paulo SP Brazil Hospital do Servidor Púbico Estadual “Francisco Morato de Oliveira”, São Paulo, SP, Brazil.

**Keywords:** Hydroxymethylglutaryl-CoA reductase inhibitors, Survival rate, Prostatic neoplasms, castration-resistant, Androgen antagonists

## Abstract

**Objective:**

To evaluate whether the addition of statins to the new antiandrogens (enzalutamide or abiraterone) affects overall survival in patients with metastatic castration-resistant prostate cancer.

**Methods:**

We searched studies in English language including the keywords statins, overall survival, and metastatic castration-resistant prostate cancer, at PubMed^®^ (MEDLINE^®^), Embase and Cochrane databases.

**Results:**

A total of 195 articles were initially identified, but only four met the inclusion criteria and were selected for the meta-analysis. A total of 955 patients, 632 on the new antiandrogens only group, and 323 on the new antiandrogens + statins group, were analyzed. In all four studies the combination therapy (new antiandrogens + statin) was well tolerated, regardless of which new antiandrogens were used. Neither the type of statin nor the doses and duration of use were well specified in the studies. The combination therapy in metastatic castration-resistant prostate cancer was associated with an overall survival improvement, and a 46% reduction in death (hazard ratio of 0.54; 95%CI 0.34-0.87; p<0.01) in multivariate analysis.

**Conclusion:**

There seems to be a clinical benefit with the association of statins to the new antiandrogens in patients with metastatic castration-resistant prostate cancer, suggesting longer overall survival with no important collateral effect. However, due to fragility of the studies available in the literature, we are not yet capable of recommending this combination of drugs in the clinical practice. Further randomized prospective studies are warranted to confirm these beneficial outcomes.

## INTRODUCTION

For the last 70 years, androgen deprivation therapy (ADT) has been considered the primary treatment for advanced prostate cancer. Although the majority of cases initially exhibit a good response to ADT, almost all patients progress to metastatic castration-resistant prostate cancer (mCRPC), which is the leading cause of mortality.^(1)^Recently, novel agents for mCRPC have been developed, such as abiraterone, which inhibits residual adrenal and intratumoral androgen synthesis by blocking CYP17A activity,^(2)^ and the new generation of androgen receptors inhibitors, such as enzalutamide,^(3)^ apalutamide and darolutamide. However, even with the use of these new drugs, disease progression still occurs in the majority of patients.^(4)^

Statins are a class of drugs that reduce cholesterol in individuals with dyslipidemia, thus lowering the risk for cardiovascular disease.^(5)^ Their mechanism of action occurs by inhibiting the 3-hydroxy-3-methylglutaryl-coenzyme, an enzyme in the mevalonate pathway of cholesterol synthesis in the liver,^(4)^ playing a role on cell proliferation, inflammation, membrane organization and steroidogenesis.^(5)^

In 2006, an epidemiological study focusing on the association of statins with risk of developing prostate cancer showed no protective effect in general, although a reduced risk of metastatic or fatal prostate cancer was demonstrated.^(6,7)^Other studies have shown that statins reduce the risk of death in prostate cancer patients on ADT,^(8)^ and postpone clinical and radiological progression, compared with non-users.^(4,9)^But not all studies have shown oncological benefits of this association.^(10,11)^

## OBJECTIVE

To evaluate whether the addition of statins to the new antiandrogens (enzalutamide or abiraterone) affects overall survival in patients with metastatic castration-resistant prostate cancer.

## METHODS

This meta-analysis was reported according to the Preferred Reporting Items for Systematic Reviews and Meta-Analyses (PRISMA) systematic review article checklist. The search was performed on December 2019 using PubMed^®^ (MEDLINE^®^), Embase and Cochrane databases, and was limited to English language documents, published with no year limit. We searched for articles with the following keywords: “statins”, “survival outcome” and “metastatic castration-resistant prostate cancer”. Review articles, abstracts from conference proceedings, and case reports were excluded. Author affiliations were used to help identify studies for which data were reported in more than one publication.

**Figure 1:**
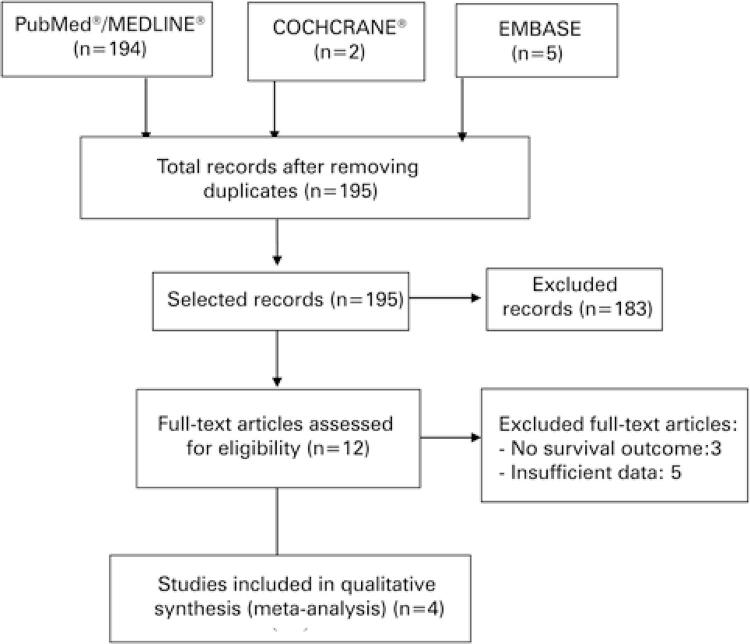
Preferred Reporting Items for Systematic Reviews and Meta-Analyses (PRISMA) Flowchart with the method of evaluating all papers with the inclusion criteria of publication in English, up to December 2019

A study was considered relevant if it compared the overall survival outcome between mCRPC patients, in use of ADT + new antiandrogens (abiraterone or enzalutamide), with or without the addition of statins. Studies were excluded if they did not fulfill these criteria. A total of 195 articles were initially identified. First, two authors reviewed the article titles to ascertain they met the inclusion criteria. Then, for relevant articles, the full text was evaluated and discrepancies were resolved via consensus, after discussion among the authors. A total of four studies were selected and presented by type of treatment (Figure 1).

The search was realized by following keywords: “statins”, “survival outcome” and “metastatic castration-resistant prostate cancer”. Review articles, abstracts of conference proceedings, and case reports were excluded. A study was considered relevant if it compared the overall survival outcome between mCRPC patients on ADT + new antiandrogens (abiraterone or enzalutamide), with or without the addition of statins. Studies were excluded if they did not fulfill these criteria. Note: This meta-analysis was reported according to the PRISMA systematic review article checklist.

Both fixed and random effects models have been performed to estimate effects. In this case, the random effects model seemed more appropriate, since we have combined studies with the same aim, which did not share the same design. The test for heterogeneity examined the null hypothesis and assumed that all studies were evaluating the same effect. The inconsistency (i^2^) describes the percentage of total variation across studies that were due to heterogeneity rather than chance. Heterogeneity among studies was analyzed by means of standard χ^2^ tests. Assessment of publication bias was done using a funnel plot, based on rank correlation.

The statistical analysis was performed using the computing environment R Statistical Software. Tables and graphs were made with both Microsoft Excel and R. The group treated with the new antiandrogens only, named No Statins, was considered the Control Group. The interventional group, treated with the combination therapy, was named Statins.

## RESULTS

The primary outcome of this meta-analysis was overall survival. Data regarding detailed inclusion criteria, results achieved and limitations were abstracted from each study individually and are described in table 1.

**Table 1:** Baseline characteristics of the studies included in the review

Reference	Location	Design	Median age (years)	Follow-up	Group (n)		Patients on study (n)

					Statins	No statins	
Boegemann et al.,^(12)^	Germany	Retrospective	70-71	2010-2015	21	87	108
Gordon et al.,^(13)^	Canada/Italy	Retrospective	72-74	2001-2016	199	399	598
Di Lorenzo et al.,^(14)^	Italy	Retrospective	65.9-67.5	2011-2016	71	114	185
Henriquez Lopez et al.,^(15)^	Spain	Retrospective	67.9-69.8	2009-2018	32	32	64
Total					323	632	955

Boegemann et al.^(12)^conducted a retrospective study of patients with metastatic prostate cancer, evaluated between 2010 and 2015. Patients were included if they had disease progression demonstrated radiographically, clinically, and/or biochemically while on standard ADT, and abiraterone before or after chemotherapy. Two cohorts were compared and analyzed for progression-free survival (PFS) and overall survival: Group 1 with 21 patients (19%) on abiraterone + ADT and statins, and Group 2 with 87 (81%) patients on abiraterone + ADT, with no statins. Significant differences were observed between the groups: patients in Group 1 had higher baseline median alkaline phosphatase levels, and a larger proportion of low-density lipoprotein (LDH)-levels above the upper limit of normal. The Gleason score (GS) ≥8 was observed in 66.7% of patients in Group 1, and in only seven patients (33.3%) in Group 2.

The median overall survival for non-statins users was 18 months, as compared to 14 months for statin users. In both univariate and multivariate analyses, concomitant use of statins was not associated with improved OS (hazard ratio – HR 1.20; 95%CI 0.7-2.1; p=0.63) and PFS (HR 1.10; 95%CI 0.6-1.8; p=0.83). A major shortcoming in this study was the great difference between the groups, which may have led to negative results.

Gordon et al.^(13)^ performed a retrospective multicenter study of patients who received abiraterone or enzalutamide for mCRPC, between January 2011 and January 2016. Patients were included in the study if they had histological confirmed prostate cancer, radiographic progression according to the Prostate Cancer Working Group 2 criteria (https://www.med-iq.com/files/cme/chapter/text-basedSuppliments/sa248/table1.html), and were treated with, at least, one D28 cycle of abiraterone or enzalutamide after previous docetaxel therapy.

Approximately one-third of the study population (199/598 patients) received statins during treatment. Of those 199 patients, 107 patients were given atorvastatin, but the statin type was not specified for all others. The median overall survival for the statins users was 20.8 months compared to 12.9 months for non-statin users, resulting in a 53% reduction in risk of death, for the patients on combination therapy (HR 0.47; 95%CI 0.35-0.63; p<0.001). Median cancer specific-survival (CSS) also significantly improved with a 57% reduction in risk of death for statin users (HR 0.43; 95%CI 0.32-0.58; p<0.001). The limitation of the study is the large number of missing data.

Di Lorenzo et al.^(14)^ carried out a retrospective study between September 2001 and January 2016, and included 187 patients with documented mCPRC, treated with at least one D28 cycle of abiraterone. All patients had histologically confirmed prostate cancer and testosterone levels of <50ng/dL.

There were 71 patients on combination therapy and 114 patients who did not receive statins. No significant difference was observed between the two groups. The exception was the percentage of GS ≥8, which was half the percentage for the group on combination therapy as compared to the non-statin users (approximately 25% *versus* 50%; p=0.001). The median overall survival for the statins users was 22.2 months compared to 15.3 months for the Non-statin Group. In both univariate and multivariate analyses, the use of statins in addition to the new antiandrogens promoted a reduction in the risk of death (HR 0.51; 95%CI 0.37-0.72; p<0.001; HR 0.40; 95%CI 0.27-0.59; p<0.001, respectively).

The limitations of this study were the lack of data for previous exposure to statins, type of statin used, treatment duration, and comorbidities. Another fact worth mentioning is the higher percentage of GS ≥8 in the Non-statin Group which may have influenced the findings.

Henriquez Lopez et al.^(15)^ performed a retrospective study between April 2009 and August 2018, and reported 64 patients with histologically confirmed mCRPC, treated with abiraterone (42 patients) or enzalutamide (22 patients) with no previous chemotherapy. The patients were equally divided into two groups (statin users *versus* non-users). No significant demographic differences were observed between the groups.

The median overall survival and PFS were 43.2 and 18.0 months for the Statin Group and 29.7 and 7.0 months for the Non-Statin Group, respectively. On multivariate analysis, the authors found the concomitant use of statins promoted a 64% reduction in risk of death (HR 0.36; 95%CI 0.13-0.99; p=0.045). Limitations of the study were missing data for some of the analyzed factors, including type of statin used, as well as prescribed doses.

Considering that all four studies assessed similar variables in relation to overall survival, the multivariate Cox-regression hazards model was used for analysis (Table 2). An i^2^ value of 74% was achieved, indicating the results between studies cannot be explained by chance. The study by Henriquez Lopez et al.,^(15)^ lacks data for the calculation of the univariate Cox regression, which is the reason for its absence in the second analysis.

**Table 2:** Overall survival in studies included

Reference	Overall survival		Population in multivariate analysis (number)

	Statins (yes *versus* no)	p value	
	HR (95%CI)		
Boegemann et al.,^(12)^	1.20 (0.7-2.1)	0.63	108
Gordon et al.,^(13)^	0.47 (0.35-0.63)	<0.001	387
Di Lorenzo et al.,^(14)^	0.40 (0.27-0.59)	<0.001	128
Henriquez Lopez et al.,^(15)^	0.36 (0.13-0.99)	0.045	64

HR: hazard ratio; 95%CI: 95% confidence interval.

**Figure 2:**
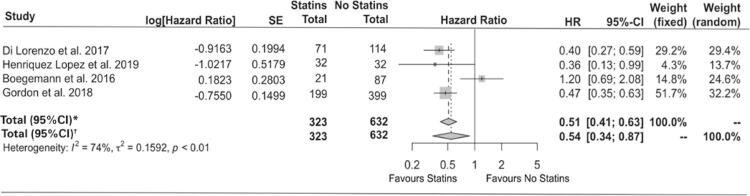
Effects of combined use of statins for treatment of castration-resistant prostate cancer on overall survival – multivariate analysis. Summary forest plot of the studies included with a multivariate analysis

**Figure 3:**
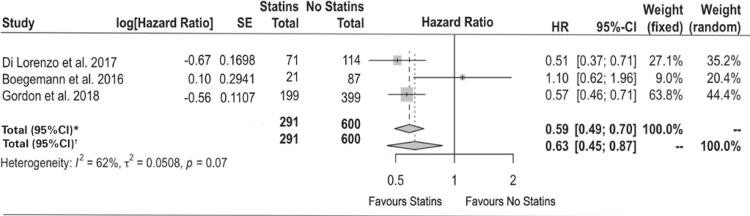
Effects of combined use of statins for treatment of castration-resistant prostate cancer on overall survival – univariate analysis. Summary forest plot of the studies included with a univariate analysis

This meta-analysis indicates the combination therapy for mCRPC had a positive effect on overall survival in a multivariate analysis (HR 0.54; 95%CI 0.34-0.87; p<0.01), when compared to patients receiving only abiraterone or enzalutamide. In the univariate analysis, in which one study had to be excluded, the same tendency was observed, but the results did not achieve statistical significance (HR 0.63; 95%CI 0.45-0.87; p=0.07) (Figures 2 and 3). In all four studies, the association of statins to enzalutamide or abiraterone was well tolerated, regardless of which new antiandrogens were used. No important collateral effect was reported.

HR: hazard ratio; 95%CI: confidence interval 95%.

HR: hazard ratio; 95%CI: confidence interval 95%.

## DISCUSSION

Statins have demonstrated a beneficial effect on prostate cancer, especially in aggressive phenotypes.^(9)^The use of statins has been shown to delay the progression of cancer among patients under ADT therapy.^(4)^ In a meta-analysis of six studies evaluating patients with localized prostate cancer, treated with surgery or radiation therapy, the use of statins was associated with a 22% reduction in risk of metastases, and a 24% reduction in risk of death due to cancer.^(16)^

Anderson-Carter et al. assessed the oncologic outcomes of 87,346 patients from the National Veterans Affairs database with advanced prostate cancer on conventional ADT. This study had 53,360 patients on statins, and 33,986 who were non-users. The Statin Group had higher Charlson comorbidity index and more cases of GS ≥8. Despite these differences, statin users had overall survival 34% longer (median 6.5 *versus* 4.0 years) and CSS 44% better than non-statin users.^(17)^

Harshman et al. evaluated castration-resistant prostate cancer (CRPC) patients treated with ADT + abiraterone, divided into users or non-users of statins. The study endpoint was duration of response to abiraterone. Patients for the study came from two major centers: Johns Hopkins University and Dana-Farber Cancer Institute. Not all patients had mCRPC. On the one hand, the Dana-Farber Cancer Institute patients on statins had a significantly longer response to abiraterone (14.2 months *versus* 9.2 months) than those without statins. On the other hand, no difference was observed for the Johns Hopkins University patients where both groups had a median of 8.0 months response to abiraterone. These controversial results may be partly explained because not all patients analyzed were metastatic, as well as for using response to abiraterone and not overall survival as the endpoint.^(18)^

It is not yet clear at what stage of the disease the use of statins promotes the greatest benefit, and there is a lack of clinical trials assessing mCPRC. The few studies available are retrospective and heterogeneous. Our meta-analysis demonstrated an overall survival 37% longer in univariate, and 46% longer in multivariate analyses, with the combination therapy for mCPRC.

Several molecular mechanisms have been implicated in the progression of CRPC, and the majority is associated with the androgen receptors signaling axis, involving androgen receptors amplification, mutations, coregulators, activation, aberrant post-translational modification and alternative splicing.^(19-22)^ Statins may work synergistically with ADT by lowering cholesterol, decreasing availability of the major substrate for androgen synthesis, thus promoting downregulation of the androgen receptors via proteolysis, altering cell-signaling pathways and inducing apoptosis of proliferating cells.^(6)^ In vitro, statins can prevent cancer progression by avoiding prostate cancer tissue to actively produce cholesterol leading to the inhibition of cell growth.^(23,24)^

Dehydroepiandrosterone sulfate is a precursor of more potent androgens and competes with statins to be transported into the cytosol of the cell using the organic anionic transporter SLCO2B1.^(25)^Thus, the pharmacodynamic interactions of statins with new antiandrogens agents, may occur either by the inhibitory effect of the residual adrenal and intratumoral androgen synthesis by blocking CYP17A, as performed by abiraterone,^(2)^ or by directly inhibiting the androgen receptors activity, as performed by enzalutamide.^(3)^

Cav-1 expression in prostate tumors has been shown to be an independent risk factor for the occurrence of CRPC, and is associated with a shorter recurrence-free survival time in these patients.^(26)^ Simvastatin was shown to augment the anticancer effects of androgen receptors antagonists by downregulating the expression of Cav-1. These findings provide evidence that Cav-1 could be a promising predictive biomarker for CRPC, and that lowering cholesterol levels with simvastatin or interfering with the expression of Cav-1 may prove to be a useful strategy to treat CRPC associated with androgen receptors inhibitors.^(26)^

Therefore, the addition of statins to the new antiandrogens agents may provide a potential new combination treatment in mCRPC, which targets different levels of the androgen receptors signaling machinery.^(2)^ This association was well tolerated and showed no important collateral effects, regardless of the use of enzalutamide or abiraterone. Additionally, statins play an important role in the primary prevention of cardiovascular events,^(27)^ especially for prostate cancer patients on ADT; and may add other protective effects, such as decrease the risk of dementia in diabetic CRPC patients.^(28)^Since statins are used worldwide, this association does not add significantly to the costs.^(27)^

A limitation of our meta-analysis is the studies included herein are all retrospective and heterogenous and, consequently, do not present the best levels of evidence for patient care. Also, the studies account neither for which type of statin (lipophilic or hydrophilic) was utilized, nor for the timing of use. Furthermore, prescribed doses were usually not specified. This raises the question of whether different statins, as well as doses and duration of treatment could promote other results.

## CONCLUSION

There seems to be a clinical benefit with the association of statins to the new antiandrogens in patients with metastatic castration-resistant prostate cancer, suggesting longer overall survival with no important collateral effect. However due to the fragility of the studies available in the literature, we are not yet capable of recommending this combination of drugs in clinical practice. Further randomized prospective studies are warranted to confirm these beneficial outcomes.
